# Artificial intelligence and precision nursing in the operating room: transforming perioperative safety and surgical outcomes

**DOI:** 10.3389/fbioe.2026.1749600

**Published:** 2026-04-10

**Authors:** Li Gong, Xia Zhao, Huana Zong

**Affiliations:** Department of Anesthesiology, The Ninth Medical Center of Chinese PLA General Hospital, Beijing, China

**Keywords:** artificial intelligence, digital transformation, perioperative nursing, predictive analytics, simulation-based education

## Abstract

**Background:**

The operating room represents a high-risk clinical environment where perioperative nurses play a critical role in patient safety, workflow coordination, and surgical outcomes. Recent advances in artificial intelligence (AI) and precision health technologies offer new opportunities to enhance perioperative nursing practice; however, the scope, maturity, and clinical relevance of these applications remain heterogeneous.

**Objective:**

This scoping review aimed to map existing evidence on the integration of AI and precision nursing in perioperative and operating room settings, with a focus on nursing roles, clinical applications, outcomes, and implementation challenges.

**Methods:**

A scoping review was conducted in accordance with PRISMA-ScR guidelines. A systematic search of PubMed/MEDLINE, Scopus, Web of Science, and CINAHL was performed for studies published between January 2010 and March 2025. Eligible studies examined AI-enabled or technologies relevant to perioperative nursing practice across the preoperative, intraoperative, and postoperative phases. Data were descriptively charted and synthesized to identify key application domains, evidence levels, and research gaps.

**Results:**

A total of 72 studies met the inclusion criteria. The AI applications identified included predictive analytics for perioperative risk stratification, real-time intraoperative monitoring and decision support, computer-vision-based workflow and sterility surveillance, automated documentation, robotic assistance, and postoperative remote monitoring. Evidence suggests potential benefits in early complication detection, workflow efficiency, and support for nursing decision-making. However, most studies were pilot or observational in nature, with limited large-scale clinical validation. Ethical considerations, data governance, workforce readiness, and integration into nursing workflows emerged as recurring challenges.

**Conclusion:**

AI-enabled precision nursing represents a promising approach to enhancing perioperative safety and surgical outcomes. Current evidence supports AI as a complementary tool that augments, rather than replaces, clinical judgment and nursing expertise. Further high-quality nursing-focused research, standardized evaluation frameworks, and ethically grounded implementation strategies are required to support safe and sustainable integration into perioperative practice.

## 1 Introduction

The operating room (OR) is a complex ecosystem that demand seamless coordination among healthcare surgeons, anesthesiologists, nurses, and technicians and is a very high-risk environment in the healthcare system where every decision can influence patient health outcomes (Pasquer et al., 2024). ORs continue to face challenges such as intraoperative errors, delays in decision-making, lapses in communication, and unpredictable patient responses to treatment. These problems arise from the need for precision, efficiency, and safety during surgical interventions (Adams et al., 2023). Recent studies have been conducted to support this shift, such as comfort nursing combined with targeted OR nursing in colorectal cancer surgery (2022–24 retrospective study), which showed shorter times to first flatus, shorter hospital stays, reduced stress markers (IL-6 and CRP), and higher patient satisfaction (Wiseman et al., 2022).

### 1.1 Precision nursing: concept and relevance in the operating room

Much research has evaluated the concept and relevance of precision nursing in the OR. Such precision nursing starts with the training and competence of OR nurses, which has been shown to improve outcomes. A systematic review found that stimulation and technology-based training improved the skills and confidence of OR nurses (Eades et al., 2023). Precision nursing has emerged as a transformative approach for integrating individualized patient data, evidence-based protocols, and predictive analytics to tailor perioperative care (Carter et al., 2014). It usually transcends a “one-size-fits for all” model by taking into consideration the physiological, environmental, and behavioral variables that influence patients’ recovery and risk of surgery. Another study demonstrated that integrating patient-specific genomic and hemodynamic data allowed perioperative nurses to anticipate adverse responses to anesthesia, leading to a 27% reduction in intraoperative complications. It also helps in the management of protocols implemented by perioperative nurses, which have been shown to reduce blood loss and postoperative infection, improve surgical recovery time, and reduce hospital stays (Abbou et al., 2022; Xu et al., 2013).

Artificial intelligence (AI) has been incorporated into this model to transform perioperative care by offering real-time data interpretation and predictive modeling (Birkhoff et al., 2021). Streams of data are analyzed in AI-driven systems via sources such as monitors, imaging, and electronic health that records to support clinical decision-making. In addition, researchers have created AI algorithms which are effective in predicting the occurrence of intraoperative hypotension prior to its onset, enabling nurses and anesthesiologists to act in advance (Lin and Lin, 2021). Similarly, AI models created through computer vision have been used to track workflows in ORs as well as to identify non-conformance with established procedures to ensure adherence to safety protocols (Gantz et al., 2019).

During the perioperative period, AI-enabled precision nursing helps enhance the final results of surgery by identifying the physiological conditions of deterioration ahead of time, dosage-optimizing anesthesia, and customizing postoperative care plans (Fecso et al., 2018). AI enables nurses to make better choices, eliminate complications, and guarantee patient-centered needs that may be fulfilled at all the levels of preoperative evaluation, intraoperative care, and postoperative treatment with the use of machine learning algorithms, robotics, and data analysis (Eckmann et al., 2022). The results of a recent multicenter trial were that AI-assisted perioperative monitoring systems reduce cases of surgical-site infections and unplanned ICU admission by approximately 35%, indicating the actual benefits of using this technology–nursing synergy (Table 1).

**TABLE 1:** Key perioperative nursing roles and associated safety/outcome domains.

S. No.	Role	Description	Associated safety	Reference
1	Preoperative assessment nurse	Screens, educates, and organizes patients for surgery	Identification of risk factors (comorbidities, allergies, and positioning risks), patient preparation, and informed consent	Xue et al. (2022)
2	Circulating nurse (intraoperative)	Coordinates OR flow, supports the surgical team, and monitors environment	Instrument count accuracy, time-outs, sterile field maintenance, and positioning/pressure injury prevention	Fecso et al. (2018)
3	Scrub nurse	Assists surgeon with instruments, maintains sterility, and anticipates needs	Surgical time efficiency, instrument availability, and contamination risk reduction	Wallis et al. (2023)
4	Postoperative (PACU/ward) nurse	Monitors recovery, early mobilization, pain control, and discharge planning	Length of stay and complications (e.g., SSI, DVT, and pneumonia) and patient satisfaction	McLean et al. (2023)

In this review article, we aim to provide a comprehensive exploration of how AI and precision nursing intersect in the OR to transform perioperative safety, reduce the burden of nurses, and improve surgical outcomes. Furthermore, this review will explore the conceptual framework of precision nursing, the role of AI technologies in the perioperative safety and care, current applications, research findings, and future directions for integrating AI-driven support into surgical nursing practice. This review also seeks to demonstrate how AI-powered precision nursing can redefine perioperative safety standards and enhance the quality of patient-centered surgical care. Next, we will examine how precision nursing frameworks are being conceptualized and applied in the surgical setting. Then, we will discuss evidence from recent studies linking precision nursing interventions to procedural safety and surgical outcomes. Finally, we will consider implementation challenges, organizational and human issues, and propose a roadmap for embedding precision nursing in the OR environment.

## 2 Methods

### 2.1 Review design

This is a scoping review that comprehensively maps the existing evidence, key concepts, and research gaps related to the application of artificial intelligence (AI) and precision nursing in perioperative and operating room settings. A scoping review methodology was considered appropriate given the emerging, heterogeneous, and multidisciplinary nature of this field, where study designs, interventions, and outcome measures vary widely.

The review was conducted and reported in accordance with “Preferred Reporting Items for Systematic Reviews and Meta-Analyses Extension for Scoping Reviews” (PRISMA-ScR) guidelines (Tricco et al., 2018).

### 2.2 Information sources and search strategy

#### 2.2.1 Search design and rationale

To ensure transparency and reproducibility in accordance with PRISMA-ScR recommendations, the exact database-specific search strategies we executed are reported below, including full queries, search fields, filters/limits, and search dates.

#### 2.2.2 PubMed/MEDLINE (National Library of Medicine)

Database: PubMed/MEDLINE
Search date: 31 March 2025
Time frame: 1 January 2010 to 31 March 2025
Language: English
Species: Humans
Article type: Journal articles
Search fields: MeSH Terms; Title/Abstract

Exact search string (as executed): (“Artificial Intelligence”[Mesh] OR “Machine Learning”[Mesh] OR “Predictive Analytics”[Mesh] OR “Artificial Intelligence”[Title/Abstract] OR “Machine Learning”[Title/Abstract] OR “Deep Learning”[Title/Abstract] OR “Predictive Analytics”[Title/Abstract] OR “Computer Vision”[Title/Abstract])

AND

(“Perioperative Nursing”[Mesh] OR “Operating Room Nursing”[Mesh] OR “Perioperative Care”[Mesh] OR “Operating Rooms”[Mesh] OR “perioperative nursing”[Title/Abstract] OR “operating room nursing”[Title/Abstract] OR “surgical nursing”[Title/Abstract])

AND

(“Intraoperative Care”[Mesh] OR “Postoperative Care”[Mesh] OR “preoperative”[Title/Abstract] OR “intraoperative”[Title/Abstract] OR “postoperative”[Title/Abstract])

Filters applied:

• Publication dates: 1 January 2010–31 March 2025
• Language: English

##### 2.2.2.1 Scopus (Elsevier)

Database: Scopus
Search date: 31 March 2025
Document types: Article; Review
Language: English
Search fields: TITLE–ABS–KEY

Exact search string (as executed):

TITLE–ABS–KEY

((“artificial intelligence” OR “machine learning” OR “deep learning” OR “predictive analytics” OR “computer vision”)

AND

TITLE–ABS–KEY

(“perioperative nursing” OR “operating room nursing” OR “surgical nursing”)

AND

TITLE–ABS–KEY

(“perioperative care” OR “intraoperative” OR “postoperative” OR “operating room”)

AND PUBYEAR >2009

AND PUBYEAR <2026

AND (LIMIT-TO (LANGUAGE, “English”))

AND (LIMIT-TO (DOCTYPE, “ar”) OR LIMIT-TO (DOCTYPE, “re”))

##### 2.2.2.2 Web of Science Core Collection (clarivate analytics)

Database: Web of Science Core Collection
Indexes searched: SCI-EXPANDED, SSCI
Search date: 31 March 2025
Language: English
Document types: Article; Review
Search fields: Topic (TS = Title, Abstract, Author Keywords, Keywords Plus)

Exact search string (as executed):

TS = (

(“artificial intelligence” OR “machine learning” OR “deep learning” OR “predictive analytics” OR “computer vision”)

AND

TS = (“perioperative nursing” OR “operating room nursing” OR “surgical nursing”)

AND

TS = (“perioperative care” OR “intraoperative” OR “postoperative” OR “operating room”)

Refined by:

• Languages: English
• Publication years: 2010–2025
• Document types: Article OR Review

Limits applied:

- Peer-reviewed journals
- English language
- Publication years: 2010–2025

No restrictions were applied based on geographic location or healthcare system.

##### 2.2.2.3 CINAHL (EBSCOhost) search strategy

Database: CINAHL Complete (EBSCOhost)
Platform: EBSCOhost
Search date: 31 March 2025
Time frame: 1 January 2010 – 31 March 2025
Language: English
Population: Humans
Publication type: Peer-reviewed journal articles
Search fields: CINAHL Headings (MH); Title (TI); Abstract (AB)

##### 2.2.2.4 Exact search string (as executed)

(MH “Artificial Intelligence+” OR MH “Machine Learning+” OR MH “Predictive Analytics” OR MH “Clinical Decision Support Systems+” OR TI (“artificial intelligence” OR “machine learning” OR “deep learning” OR “predictive analytics” OR “computer vision”)

OR AB (“artificial intelligence” OR “machine learning” OR “deep learning” OR “predictive analytics” OR “computer vision”)

AND

(MH “Perioperative Nursing” OR MH “Operating Room Nursing” OR MH “Perioperative Care” OR MH “Operating Rooms” OR MH “Surgical Nursing” OR TI (“perioperative nursing” OR “operating room nursing” OR “surgical nursing”) OR AB (“perioperative nursing” OR “operating room nursing” OR “surgical nursing”)

AND

(MH “Preoperative Care” OR MH “Intraoperative Care” OR MH “Postoperative Care” OR TI (preoperative OR intraoperative OR postoperative OR “operating room”) OR AB (preoperative OR intraoperative OR postoperative OR “operating room”)

##### 2.2.2.5 Limits applied

- Publication dates: January 2010 – March 2025
- Language: English
- Peer-reviewed: Yes
- Species: Humans

### 2.3 Eligibility criteria

#### 2.3.1 Inclusion criteria

Studies were included if they:

- Focused on AI-enabled or data-driven technologies relevant to perioperative or operating room nursing.
- Addressed nursing roles, decision-making, workflow, safety, or patient outcomes.
- Included empirical studies (quantitative, qualitative, or mixed methods), pilot studies, implementation studies, or high-quality reviews.
- Were published in peer-reviewed journals.
- Were written in English.

#### 2.3.2 Exclusion criteria

Studies were excluded if they:

- Focused exclusively on surgeon- or anesthesia-only AI applications without nursing relevance.
- Addressed AI in healthcare without a perioperative or surgical context.
- Were editorials, opinion pieces, conference abstracts without full text, or non-peer-reviewed reports.
- Lacked sufficient methodological or outcome-related information.

### 2.4 Study selection process

All retrieved records were exported to reference management software, and duplicate entries were removed prior to screening. The selection process followed a two-stage approach.

1. Title and abstract screening was performed to assess relevance based on the predefined eligibility criteria.
2. Full-text screening was subsequently conducted for all potentially eligible articles.

Screening was performed independently by two reviewers, with discrepancies resolved through discussion and consensus. When necessary, a third reviewer was consulted to resolve disagreements.

### 2.5 Data charting and synthesis

Data from included studies were charted using a standardized extraction framework capturing:

- Study characteristics (author, year, country, and design).
- Perioperative phase (preoperative, intraoperative, and postoperative).
- AI technology or digital system applied.
- Nursing role and level of clinical involvement.
- Reported outcomes related to safety, efficiency, decision-making, or patient care.
- Key limitations and implementation challenges.

The findings were descriptively and thematically synthesized, with results organized according to perioperative phases and AI application domains, consistent with scoping review methodology.

### 2.6 Results of study selection

The database search yielded 1,248 records across PubMed/MEDLINE, Scopus, Web of Science, and CINAHL. After removing 312 duplicate records, 936 records remained for title and abstract screening. Of these, 821 were excluded due to irrelevance to perioperative nursing, absence of AI-related applications, or lack of empirical or review-level evidence.

A total of 115 full-text articles were assessed for eligibility. Following full-text evaluation, 72 met the predefined inclusion criteria and were included in the final synthesis. Excluded full-text articles were primarily removed due to limited nursing relevance, insufficient methodological detail, or a focus on surgical or anesthetic AI applications without perioperative nursing involvement.

**FIGURE 1:**
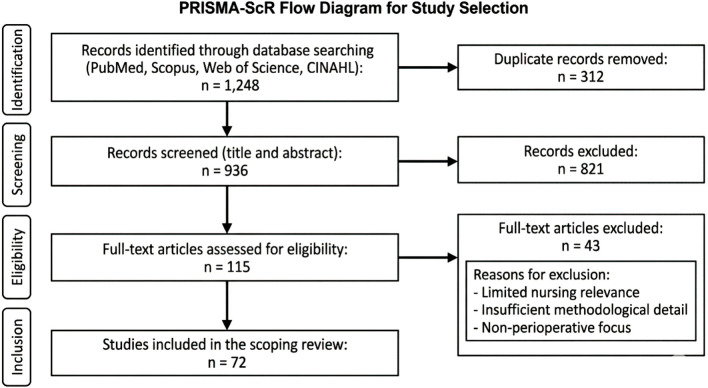
PRISMA-ScR flow diagram for study selection.

The study selection process is illustrated in Figure 1.

### 2.7 PRISMA-ScR flow diagram

A PRISMA-ScR flow diagram was developed to transparently depict the identification, screening, eligibility, and inclusion of studies at each stage of the review process, in accordance with PRISMA-ScR reporting standards.

## 3 Integration of AI and precision nursing across perioperative phases

Precision nursing across the perioperative, preoperative, intraoperative, and postoperative stages marks a gradual evolution in particular surgical care. After the integration of analytical approaches and assessment, perioperative nursing transitions from a generalized to a patient-specific framework, most importantly in optimizing surgical outcomes, enhancing patient safety, and improving efficiency through data-informed clinical practices (Abbou et al., 2022). In the initial stage, apparently, precision nursing emphasizes risk probability and optimization for patients before surgery. Data derived from the assessments, laboratory parameters, and patient-reported outcomes are analyzed to predict risks, particularly from anesthesia intolerance, potential for bleeding, and delayed recovery (Senol et al., 2023).

In the preoperative phase, studies have demonstrated that AI-driven predictive analytics synthesize patient demographics, comorbidities, and surgical risk data to stratify patients and anticipate complications such as infection, bleeding, or delayed recovery (Hilary and Lisa, 2005). This also allows nurses to tailor pre-surgical education, optimization, and preparation of protocols and also effectively allocate resources to health outcomes (Hannah Ji et al., 2022). However, another study followed AI’s utility in identifying high-risk of surgical candidates, which can improve triage accuracy and support evidence-based nursing intervention. In the next stage, the intraoperative phase, this AI system interfaces with monitoring equipment and manages electronic health records (EHRs) to deliver real-time alerts in physiological deviations, equipment malfunctions, or workflow inefficiencies (Theijse et al., 2023). Continuous data flow empowers the perioperative phase in which nurses anticipate hemodynamic instability, temperature fluctuations, and anesthetic depth variations, thus ensuring timely intervention and improved team coordination (Jia et al., 2017).

In the last stage, the postoperative, AI aids in patient monitoring, pain assessment, wound evaluation, and early detection of complication through sensor-based systems and algorithm alerts. It predictively assists nurses identify patients at risk of readmission or infection, enables earlier interventions, and individualizes discharging planning (Jonathan et al., 2018). Moreover, AI supports the postoperative handoffs, which ensures and establishes the continuity of care and minimizes communication lapses during critical transitions. As well as direct patient management, AI optimizes the perioperative workflow and resource utilization, reduces the documentation burden, and enhances the nurse’s productivity (Tuwatananurak et al., 2019). By automizing administrative tasks such as scheduling, supply management, and case sequencing, AI allows researchers to focus on more high-value and patient-centered activities. Table 2 integrates the conceptual model of precision nursing and AI across the perioperative phases.

**TABLE 2:** Integration of precision nursing and AI across the perioperative phases.

S. No.	Perioperative phase	Precision nursing focus	AI-based application	Outcome improvement	Reference
1	Preoperative	Risk assessment, optimization, and anxiety reduction	Predictive models using EHR and laboratory data for surgical readiness	Reduced cancellations and improved preoperative optimization	Lauren et al. (2023)
2	Intraoperative	Real-time monitoring and coordination	Data integration from anesthesia systems, hemodynamic sensors, and imaging	Early detection of instability and fewer intraoperative errors	Xue et al. (2021)
3	Postoperative	Pain control, wound healing, and infection prevention	Predictive analytics and wearable monitoring for recovery tracking	Reduced complications and enhanced recovery outcomes	Jeffrey et al. (2023)

**FIGURE 2:**
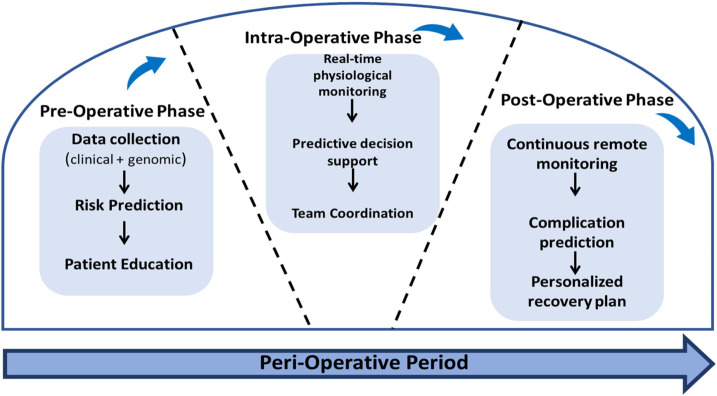
Framework of precision nursing integration across perioperative phases.

AI integrates collectively into precision nursing, which represents a paradigm shift from reactive to proactive care and thus transforms perioperative nursing practice into a model that is anticipatory, personalized, and data-informed (McLean et al., 2021). In early evidence, this highlights promising outcomes in operational efficiency and patient safety as well as situational awareness. However, further large-scale clinical studies are essential to validate AI’s long-term impact on surgical outcomes and nursing performance metrics (Kirollos et al., 2018). The integration of precision nursing within the three phases can be conceptualized as a continuous, data-driven cycle that encompasses three interconnected phases: preoperative, intraoperative, and postoperative (Figure 2). Real-time data from hemodynamic sensors, anesthesia machines, and surgical imaging tools are interpreted to maintain optimal anesthesia depth, fluid balance, and sterile compliance.

### 3.1 Data-driven decision-making and clinical judgment

The notion of data-driven and decision-support technologies is one of the key tenets of precision nursing in the OR, and in harmony with human clinical judgment it produces large and dynamic datasets formed of anesthesia monitors, EHRs, and intraoperative imaging systems (Abbou et al., 2022). Along with these developments is the challenge of making such information context-specific and more useful clinical information that could improve the safety outcomes of the patients (Zhang et al., 2015; Xue et al., 2022). The incorporation of nursing experience is invaluable, since data may be used to make decisions, yet sophisticated decision-making is impossible without diverse experiences, situational knowledge, and ethical consideration (Butler et al., 2025). More specifically, when we refer to precision nursing, a symbiotic relationship between computation analysis and human interpretation is required. Cooperation with data-driven decision-making and precision nursing serves as perioperative care between a reactive or a proactive intervention for the patients (Nyanchoka et al., 2019). Table 3 denotes that data-driven systems are not substitutes for the professional intuition of nurses—they only enhance it. Human machine synergy provides greater accuracy and timeliness of interventions where ethical, empathetic, and situational awareness is crucial to patient-centered outcomes.

**TABLE 3:** Data-driven decision-making in precision nursing.

S. No.	Clinical context	Analytical method	Outcome supported	Role of nurse	Reference
1	Hemodynamic instability prediction	Cardiovascular signal analysis (ECG and BP waveforms)	Early identification of cardiac events	Interpret model output and initiate preventive care	Eades et al. (2023)
2	Surgical error detection	Image-based computer vision	Prevention of intraoperative injury	Coordinate with surgeon for corrective action	Adams et al. (2023)
3	Workflow documentation	Natural language processing	Enhanced continuity of care	Validate records and plan follow-up interventions	Nyanchoka et al. (2019)

Effective collaboration ensures better patient care, and decisions are usually made through a shared understanding of clinical goals, procedural requirements, and individual patient needs. In all steps of surgical care, nurses are central in coordinating, communicating, and monitoring patients’ responses and ensuring continuity across all surgical stages (Kai et al., 2021). In the process of decision-making for treatment, surgeons depend on nurses for the accurate diagnosis of patients for intraoperative monitoring and the readiness of instruments, while anesthesiologists rely on continuous feedback from nurses to maintain physiological stability. Nurses also depend on the timely adjustment of surgical updates and anesthetics cues to anticipate patient needs and adapt their care accordingly. This interdependence reinforces the safety culture, which is based on communication, mutual respect, and accountability. (Salm et al., 2020).

The concept of interdisciplinary integration in perioperative nursing is increasingly identified as a fundamental principle of precision-based practice. The implementation of lean documentation systems, uniformity of perioperative checklists, and common communication dashboards enhances correct information and minimizes procedural errors (Schiff et al., 2016). It has been proven by many studies that the use of structured communication tools, including perioperative briefings, safety huddles, and standard handoff protocols are very effective in reducing the rate of adverse events and improving the intraoperative rate. Moreover, the use of the World Health Organization (WHO) surgical safety checklist has been reported to decrease the incidence of postoperative complications and mortality by 30% in a multicenter study, which highlights the role of standardized communication in surgical outcomes (Maria et al., 2019; Persoon et al., 2011). The interdisciplinary interaction of precision nursing as a conceptual framework in Figure 2 represents the cycle and adaptive process in which communication informs assessment and directs intervention and in which evaluation feeds back into the team learning and workflow optimization (Schiff et al., 2016).

Currently, the incorporation of a coordination platform and an AI-powered system that consolidates nurse, anesthetist, and surgeon workflows is also highlighted in emerging studies, decreasing irregularity in data-based support and perioperative communication. The collaboration, interdisciplinarity, and integration of the working procedure is the backbone of precision nursing in the perioperative units (Schiff et al., 2016). It fuses the professional experience of collaborating communication amongst healthcare teams, providing safe, more efficient, and personalized surgical care to support both technical and humanitarian values. Table 4 conveys significant AI implementation in perioperative nursing care and its clinical implications.

**TABLE 4:** Major AI applications in perioperative nursing and their clinical implications.

S. No.	AI domain	Description	Examples in practice	Nursing implication	Clinical outcome	Reference
1	Predictive analytics	Uses large-scale patient and surgical data to identify risk factors for complications such as hypotension, bleeding, infection, or delayed recovery	Risk prediction tools in anesthesia monitoring and postoperative complication forecasting systems	Enables proactive patient optimization, early risk identification, and tailored preparation	Reduced intraoperative events and improved postoperative stability	Fecso et al. (2018)
2	Image and video analysis	Real-time interpretation of surgical video feeds and imaging data to track workflow accuracy and maintain sterility	Surgical workflow mapping and contamination detection systems	Enhances situational awareness and reduces human error during critical steps	Improved safety and adherence to sterile protocols	Martinez et al. (2021)
3	Automated documentation	Transforms spoken or written clinical notes into structured formats for perioperative records	Voice-based operative reports and real-time transcription software	Minimizes documentation time and improves record consistency	Streamlined workflow and better communication continuity	Tuwatananurak et al. (2019)
4	Robotic assistance	Provides automated or semi-automated support for surgical preparation and instrument handling	Robotic scrub nurses anticipating instrument needs and sterilization robots	Decreases manual workload and enhances procedural accuracy	Improved efficiency and reduced procedural time	Schiff et al. (2016); Kanabolo et al. (2024)
5	Clinical decision support systems (CDSS)	Integrates multimodal data to guide intraoperative and postoperative interventions	Integrated anesthesia monitoring dashboards and postoperative surveillance systems	Assists in making informed clinical decisions and improves early complication and detection	Fewer preventable complications and faster recovery	Thomas et al. (2014)

## 4 Predictive analytics in perioperative precision nursing

**FIGURE 3:**
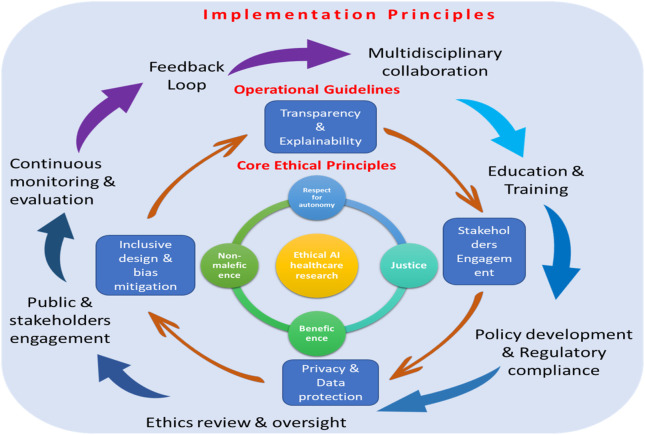
Implementation principles in operational and core ethical development.

Predictive analysis, which encompasses everything from preoperative to postoperative planning, indicates the identification of high-risk patients from analyzing past histories. Researchers plan to optimize the schedule to predict a better time for treatment, reducing delays and improving the utilization of the OR during treatment (Gabriel et al., 2022). With the help of predictive analysis, perioperative precision nursing could enable the transition from reactive decision-making toward proactive, data-driven interventions by surgeons (Zhang et al., 2024). In addition, hospitals could predict the need for blood products, ICU beds, and other resources, and utilize the model in large settings. Recent advances in machine learning (ML) and deep learning (DL) techniques have facilitated real-time risk assessment by analyzing physiologic, biochemical, and behavioral data streams, often captured through perioperative monitoring systems and EHRs (Figure 3) (Jeffrey et al., 2023).

The surgical journey does not end in the OR, so predictive models are developed for post-surgical use, such as providing real-time insights for making quick and informed decisions (Krell et al., 2014). Predictive analytics can also improve postoperative care by identifying patients at risk of complications like infection or readmission. This allows targeted interventions, such as closer monitoring or early discharge planning. These models evaluate parameters such as comorbidities, vital-sign trends, laboratory results, and intraoperative responses to estimate potential complications (Tricco et al., 2018). One major study has found that high-risk stratification systems detect the early signs of hemodynamic instability or postoperative infections with high sensitivity. Data-driven risk calculators mostly use preoperative laboratory values and patient histories, which have been successfully incorporated into anesthesia planning to improve patient optimization, reduce last-minute surgical cancellations (Krell et al., 2014), and improve the identification of high-risk diseases in order to take proactive measures to improve patient outcomes. Predictive analytics can help reduce unnecessary tests and procedures, leading to significant cost savings. Better scheduling and resource allocation mean less downtime and more efficient use of ORs and staff (Kwan et al., 2023; Siam et al., 2017). When surgeries go smoothly and recovery times are shorter, patient satisfaction naturally improves. Similarly, infection surveillance tools applied postoperatively help nurses tailor antibiotic regimens and wound-care strategies based on individual patient risk profiles (Table 5).

**TABLE 5:** AI and digital technologies in perioperative nursing practice.

S. No.	Domain	Technological advance	Nursing impact	Clinical outcome	Reference
1	Real-time monitoring and decision support in surgery	Integrated dashboards combining hemodynamics, anesthesia depth, and temperature data; automated feedback systems; intraoperative video analysis tools	Enables perioperative nurses to detect deviations quickly, anticipate patient instability, and coordinate timely interventions	Reduced hypotension, fewer intraoperative delays, improved patient stability, enhanced sterile compliance, and reduced contamination risk	Xu et al. (2013); Gillespie et al. (2019); Engelmann et al. (2014)
2	Automation and robotics in surgery	AI-driven robotic systems for instrument handling, supply management, and environmental preparation	Reduces repetitive manual tasks, allowing nurses to focus on continuous monitoring, intraoperative communication, and patient advocacy	Improved efficiency, procedural precision, and perioperative nurse satisfaction	Greenberg et al. (2017)
3	Documentation and handover	Speech-recognition and voice-to-text documentation tools; standardized handover protocols (e.g., SBAR)	Minimizes transcription errors and documentation time; improves completeness of notes and communication accuracy	Fewer adverse events, enhanced efficiency, and increased staff satisfaction	Strömblad et al. (2021); Levac et al. (2010)
4	Postoperative surveillance and follow-up	Mobile apps for wound monitoring, tele-follow-up, wearable devices tracking vitals, and activity	Enables remote nursing oversight, early detection of complications, and patient engagement in recovery	Reduced outpatient visits, earlier detection of wound or cardiorespiratory complications, and improved patient outcomes	Moris and Linos (2013); Hovlid et al. (2013)
5	Instrument and supply traceability	Barcodes, RFID systems for surgical instrument tracking; IoT-enabled sensors	Supports real-time verification of instruments and sponges; continuous monitoring of environmental and physiological parameters	Reduces retained surgical items, enhances instrument management accuracy, and predicts potential complications	Fecso et al. (2018)

### 4.1 Real-time monitoring and decision support system

The current system has become functionally essential in modern surgical practice, enhancing safety and coordination in the operating room. The more advanced versions are now equipped with clinical decisions that support capabilities that interact with end-users to facilitate prompt, safe, accurate, and informed decision-making. Using an automated feedback system to analyze intraoperative signals and maintain optimal anesthesia and balance of fluid reduces the incidence of hypotension and intraoperative delays and improves overall patient stability (York et al., 2022). For example, some preoperative evaluation modules provide a robust, electronic history-taking questionnaire and suggest preoperative laboratory tests based on algorithms that take into account specific procedures and any comorbidities for a particular patient (Xue et al., 2021).

Robotics in surgery and automation have eased the burden of repetitive manual tasks, such as instrument handling and environmental preparation, allowing nurses to prioritize high-value activities like continuous physiological monitoring, intraoperative communication, and patient advocacy (Eades et al., 2023). Consequently, AI-driven robotic assistance enhances procedural precision, efficiency, and the satisfaction of nurses. For documentation and handover, speech-recognition voice-to-text systems have demonstrated their ability to reduce transcription errors and time in documentation while improving the completeness of intraoperative records (Wiseman et al., 2022). The implementation of standardized protocols such as SBAR has consistently minimized communication gaps, contributing to fewer adverse events and higher staff satisfaction. Postoperative surveillance has also advanced through digital technologies, including smartphone-based wound monitoring and by wearable devices that transmit vital signs and activity data to nursing dashboards (Abdel Raheem et al., 2017). Such tools have proven effective in the early identification of complications and in reducing unnecessary outpatient visits. Current traceability technologies like barcoding, RFID, and IoT enable systems that help track surgical instruments and supplies in real time. These systems help prevent retained surgical items, which improves the accuracy of instrument counts, and they monitor both environmental and physiological parameters to predict potential complications (Zhang et al., 2015). These advances in workflow design and the integration of digital tools and evidence-based nursing protocols significantly improve perioperative documentation so that postoperative outcomes can be achieved.

### 4.2 Ethical and operational considerations

The shift to digitally enhanced perioperative practice presents ethical, legal, and operational issues which must be intentionally addressed and are crucial to ensuring patient trust. One issue is data governance and privacy. Collecting and transmitting clinical and patient-reported data continuously exposes the vulnerability of breaches and misuse (Eades et al., 2023). More recent surveys of digital health ethics also propose effective governance structures and compliance with local data-protection regulation, explicit informed consent to remote monitoring, and effective contractual relations with technology suppliers (Kwan et al., 2023). Multiple investigations point to the inappropriateness of deployment to clinical processes, the insufficiency of training, and the absence of technical support as the primary reasons of software failure. Technology roll-out techniques using simulation-based training, competency testing, and continual user feedback yield increased adoption and less unsafe application in perioperative environments (Barbat et al., 2020). Practical training in documentation tools, remote monitoring workflows, and the interpretation of device-generated alerts should therefore be included in nursing curricula and in-service education (de Leval et al., 2011).

Plans for implementation must thus involve cost–benefit analysis, gradual implementation, and assessment of low-cost options that would maintain safety benefits without additional burden on unsustainable spending (Jeannette et al., 2018). These policies would have to be co-designed by interdisciplinary committees (nursing, surgery, anesthesia, IT, and legal) and regularly audit outcomes in order to ensure patient safety and ethical compliance (Jia et al., 2017). AI can transform perioperative nurses to provide personal, efficient, and predictive care through synergies of predictive analytics, robots, NLP-based documentation, and intelligent monitoring devices. Technological collaboration is not a substitute human skills; instead, it enhances the judgment, empathy, and critical thinking of perioperative nurses, making them the drivers of the digital transformation of surgical care (Jonathan et al., 2018).

## 5 Evaluation, challenges, and research perspectives

This review of digital systems in the field of perioperative precision nursing shows both quantifiable gains and current difficulties. The most recent application, the Perioperative Chatbot (PEACH), has shown high accuracy in assessment and enhanced coordination, which indicates the potential of data-driven decision support tools to provide a transformative effect. Nevertheless, issues of data quality, interoperability and workforce preparedness still hinder large-scale adoption (Xue et al., 2022). There are also ethical implications, in that the technology is supposed to act as a cognitive aid that supports, but is not a substitute for, clinical judgment (Butler et al., 2025). Simultaneously, explanatory AI, privacy-sensitive learning, and the integration of multimodal data are pushing the boundaries of precision nursing. Educational interventions based on simulation with the help of virtual and augmented environments further enhance the acquisition of skills, and federated learning frames stimulate the collaborative refinement of the model without data de-anonymization (York et al., 2022). The sustainable development of digital and precision nursing is based on the clear design of systems, improved data literacy, and the incorporation of human expertise, moral responsibility and interdisciplinary cooperation (Table 6).

**TABLE 6:** Evaluation, challenges, and research perspectives in digital and precision nursing.

S. No.	Subsection	Focus area	Key finding	Challenge	Research perspective	Studies mentioned	Reference
1	Evaluating the impact of digital systems in precision nursing	Assessment of digital systems	Data-driven technologies have transformed perioperative nursing, improving accuracy, decision-making, and coordination	Data quality, heterogeneity, bias, interoperability issues, and workforce readiness gaps	Strengthen governance frameworks, enhance data literacy, and encourage multidisciplinary collaboration	Perioperative Chatbot (PEACH)—96.7% assessment accuracy; clinicians report quicker decisions and better coordination	Theoneste et al. (2022)
2		Perception and readiness	Nurses show enthusiasm toward technology-assisted care but express concerns about insufficient training and loss of autonomy	Limited digital training and apprehension about professional role changes	Continuous education and supportive transition to digital workflows	Survey among perioperative nurses in Türkiye	Hu et al. (2014)
3		Ethical and professional dimensions	Technology acts as a cognitive aid that supports clinical judgment and accountability	Risk of over-reliance on automation and insufficient human oversight	Emphasize ethical design, ensuring sustained human control and responsibility	Review of technology-assisted nursing confirms complementary role of technology	Gabriel et al. (2022)
4	Research directions and innovation pathways	Explainable and transparent systems	Visualization-driven dashboards and interpretable decision aids improve situational awareness and trust	Complexity of model interpretation and lack of standardization	Develop explainable AI frameworks for transparent decision-making	Dashboards mapping hemodynamic trends or infection risk probabilities	Tianqi et al. (2022)
5		Privacy-preserving collaboration	Federated learning enables multicenter data sharing without compromising patient privacy	Technical and regulatory challenges in implementation	Advance federated models for ethically compliant predictive analytics	Multicentre surgical studies applying privacy-preserving learning models	Yan et al. (2015)
6		Simulation-based education	Virtual and augmented reality simulations improve perioperative skills, response times, and clinical accuracy	Cost, accessibility, and scalability of simulation technologies	Integrate digital simulation into standard nursing education and skill development	Studies showing improved intraoperative management through digital simulations	Kanabolo et al. (2024)
7		Multimodal data integration	Integration of genomic, microbiome, and physiological data supports personalized perioperative risk profiling	Complexity in data integration and interpretation	Foster research in systems biology and personalized perioperative strategies	Studies linking genetic polymorphisms, nasal microbiome, and postoperative infection risk	José et al. (2021)
8		Human–technology collaboration	Active nurse engagement in interpreting system outputs enhances patient safety and workflow efficiency	Potential deskilling and dependency on automated outputs	Design systems that promote shared decision-making and ethical collaboration	Observational analyses showing improved safety and efficiency with nurse validation of system outputs	Kai et al. (2021)

## 6 Policy, education, and global implementation

**FIGURE 4:**
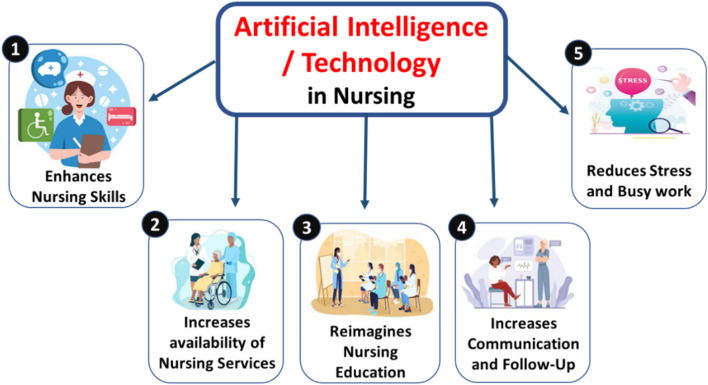
Artificial intelligence and technology effect in nursing section.

The successful integration of digital systems into perioperative nursing depends on robust policy frameworks, competency development, and international collaboration to ensure both safety and sustainability (Lundberg et al., 2020). Global health organizations, including the International Council of Nurses (ICN) and WHO, emphasize the importance of transparent validation, equitable deployment, and ethical oversight in data-driven healthcare systems (Figure 4). National and institutional policies must align with these global directives to safeguard data integrity, ensure patient privacy, and maintain accountability in AI-assisted clinical decisions (Nasiri et al., 2021).

The evolving landscape of precision and AI-enabled nursing necessitates a redefined educational framework that prioritizes digital literacy, ethical reasoning, and data interpretation skills. Nursing curricula should integrate simulation-based learning and competency-based certification programs, which have already shown promising outcomes in academic and tertiary healthcare centers (Schulz et al., 2020). These educational reforms will equip perioperative nurses with the knowledge to interpret predictive analytics, manage intelligent monitoring systems, and collaborate effectively with AI-driven tools during surgical care. International partnerships are vital to bridging disparities in access to digital health innovations. Establishing global “precision nursing consortia” can facilitate data harmonization, shared learning, and the standardization of best practices across regions (Golnar et al., 2022). Furthermore, creating centers of excellence in perioperative digital nursing will serve as dedicated hubs for interdisciplinary training, research advancement, and policy alignment (Table 7). Such coordinated efforts can accelerate the ethical, evidence-based implementation of precision nursing frameworks worldwide, ensuring that technological progress translates into equitable patient outcomes and sustainable healthcare transformation.

**TABLE 7:** Evaluation metrics for AI implementation in perioperative nursing.

S. No.	Evaluation domain	Key indicator	Example tools/Methods	Expected benefit	Reference
1	Clinical effectiveness	Prediction accuracy and reduction in intraoperative events	ROC analysis and model calibration	Improved patient outcomes	Hadiza et al. (2012)
2	Operational efficiency	Time saved and workflow adherence	Time–motion studies and EHR audit	Enhanced nursing efficiency	Rozario and Rozario (2020)
3	Human factors	Nurse acceptance, cognitive load, and alert fatigue	NASA-TLX and survey-based studies	Higher satisfaction and safety	Helena et al. (2023)
4	Ethical compliance	Data privacy and algorithm bias	Audit trails and fairness indices	Transparent, equitable care	Lam et al. (2022)
5	Educational outcomes	AI literacy and adaptation	Pre/post-training evaluations	Increased nurse competency	Louise et al. (2024)

## 7 Limitations

Several limitations should be considered when interpreting the findings of this review. First, this study was conducted as a scoping review rather than a systematic review. While this approach was appropriate for mapping the breadth of evidence in an emerging and interdisciplinary field, it does not include formal quality appraisal or meta-analysis. Consequently, the strength of evidence across the included studies was not quantitatively assessed.

Second, the literature included demonstrated substantial heterogeneity in study design, AI technologies, clinical settings, outcome measures, and levels of nursing involvement. This variability limited direct comparison across studies and precluded definitive conclusions regarding the effectiveness or clinical superiority of specific AI applications.

Third, despite growing interest in AI-enabled perioperative care, there remains a limited number of high-quality, nursing-specific clinical trials. Many of the studies included were pilot investigations, observational analyses, or proof-of-concept implementations, often focusing on technical performance rather than patient-centered or nursing-sensitive outcomes. This restricts the generalizability of findings to routine perioperative nursing practice.

Finally, publication bias may have influenced the available evidence, as studies reporting positive or innovative applications of AI are more likely to be published than those describing neutral or negative results. In addition, restriction to English language, peer-reviewed literature may have excluded relevant unpublished or non-English studies.

While AI can augment perioperative nursing by enhancing risk prediction and workflow monitoring, it does not replace the foundational benefits of workforce-centered interventions such as simulation-based training, standardized checklists, and team communication programs, which have robust evidence for improving safety and non-technical skills. Unlike training-only approaches, AI introduces distinct failure modes. Automation bias may lead nurses to over-trust algorithmic recommendations and discount conflicting clinical judgment, particularly in high-pressure operating room environments. Alert fatigue, caused by frequent or low-specificity AI notifications, can reduce responsiveness to critical warnings and increase cognitive load. Additionally, prolonged reliance on AI systems raises concerns about over-reliance and potential deskilling, whereby continuous decision support may erode independent clinical reasoning and vigilance. These risks underscore that AI should function strictly as a decision-support tool, layered onto strong human-centered safety systems, with targeted training, alert governance, and ongoing oversight to ensure safe and effective integration into perioperative nursing practice.

These limitations highlight the need for rigorously designed, nurse-led, and ethically grounded research to strengthen the evidence base and to support the safe, effective integration of AI into perioperative nursing practice.

## 8 Conclusion

The integration of AI and precision technologies into perioperative nursing marks a transformative shift in surgical safety, efficiency, and patient outcomes. Through predictive analytics, multimodal data integration, and simulation-based learning, nurses can better anticipate complications, personalize interventions, and optimize surgical workflows. However, effective clinical translation requires robust ethical governance, professional readiness, and international collaboration. Transparent governance frameworks are essential to safeguard patient autonomy, data security, and accountability across perioperative care. Policy guidance aligned with international bodies such as the International Council of Nurses and the World Health Organization can promote equitable and responsible AI adoption. Simultaneously, strengthening digital competencies through updated curricula, simulation training, and interdisciplinary education is critical to sustaining clinical excellence. Establishing international precision nursing consortia and centers of excellence can further accelerate research coordination, data harmonization, and knowledge sharing, bridging gaps between high- and low-resource systems. Ultimately, AI-driven perioperative precision nursing represents not only technological advancement but also a pathway toward safer, more intelligent, and ethically grounded healthcare.
